# INEXAS: A Phase 2 Randomized Trial of On‐demand Inhaled Interferon Beta‐1a in Severe Asthmatics

**DOI:** 10.1111/cea.13765

**Published:** 2020-11-03

**Authors:** Christopher McCrae, Marita Olsson, Per Gustafson, Anna Malmgren, Malin Aurell, Malin Fagerås, Carla A. Da Silva, Anders Cavallin, Jonathan Paraskos, Karin Karlsson, Cecilia Wingren, Phillip Monk, Richard Marsden, Tim Harrison

**Affiliations:** ^1^ Translational Science and Experimental Medicine, Research and Early Development Respiratory & Immunology, BioPharmaceuticals R&D AstraZeneca Gaithersburg Maryland USA; ^2^ Krefting Research Centre Department of Internal Medicine and Clinical Nutrition Institute of Medicine University of Gothenburg Gothenburg Sweden; ^3^ Early Biometrics and Statistical Innovation Data Science and AI, BioPharmaceuticals R&D AstraZeneca Gothenburg Sweden; ^4^ BioPharmaceutical Medical AstraZeneca Gothenburg Sweden; ^5^ Early Respiratory & Immunology Projects Department BioPharmaceuticals R&D AstraZeneca Gothenburg Sweden; ^6^ Early Respiratory & Immunology Clinical Development BioPharmaceuticals R&D AstraZeneca Gothenburg Sweden; ^7^ Translational Science and Experimental Medicine Early Cardiovascular, Renal and Metabolism (CVRM) BioPharmaceuticals R&D AstraZeneca Gothenburg Sweden; ^8^ Point of Care Diagnostics, Precision Medicine Oncology R&D AstraZeneca Cambridge UK; ^9^ Synairgen Research Ltd Southampton University Hospital Southampton UK; ^10^ Nottingham NIHR Biomedical Research Centre University of Nottingham Nottingham City Hospital Nottingham UK

**Keywords:** asthma, eosinophils, exacerbation, IFN response, IL‐18, interferon, viral URTI

## Abstract

**Background:**

Upper respiratory tract infections (URTIs) are important triggers for asthma exacerbations. We hypothesized that inhalation of the anti‐viral cytokine, interferon (IFN)‐β, during URTI, could prevent these exacerbations.

**Objective:**

To evaluate the efficacy of on‐demand inhaled IFN‐β1a (AZD9412) to prevent severe asthma exacerbations following symptomatic URTI.

**Methods:**

This was a randomized, double‐blind, placebo‐controlled trial in which patients with severe asthma (GINA 4‐5; n = 121) reporting URTI symptoms were randomized to 14 days of once‐daily nebulized AZD9412 or placebo. The primary endpoint was severe exacerbations during treatment. Secondary endpoints included 6‐item asthma control questionnaire (ACQ‐6) and lung function. Exploratory biomarkers included IFN‐response markers in serum and sputum, blood leucocyte counts and serum inflammatory cytokines.

**Results:**

Following a pre‐planned interim analysis, the trial was terminated early due to an unexpectedly low exacerbation rate. Asthma worsenings were generally mild and tended to peak at randomization, possibly contributing to the lack of benefit of AZD9412 on other asthma endpoints. Numerically, AZD9412 did not reduce severe exacerbation rate, ACQ‐6, asthma symptom scores or reliever medication use. AZD9412 improved lung function (morning peak expiratory flow; mPEF) by 19.7 L/min. Exploratory *post hoc* analyses indicated a greater mPEF improvement by AZD9412 in patients with high blood eosinophils (>0.3 × 10^9^/L) at screening and low serum interleukin‐18 relative change at pre‐treatment baseline. Pharmacodynamic effect of AZD9412 was confirmed using IFN‐response markers.

**Conclusions & Clinical Relevance:**

Colds did not have the impact on asthma patients that was expected and, due to the low exacerbation rate, the trial was stopped early. On‐demand AZD9412 treatment did not numerically reduce the number of exacerbations, but did attenuate URTI‐induced worsening of mPEF. Severe asthma patients with high blood eosinophils or low serum interleukin‐18 response are potential subgroups for further investigation of inhaled IFN‐β1a.

## 1 INTRODUCTION

Upper respiratory tract infections (URTIs) are known to be a major risk factor for asthma exacerbations. Up to 95% of asthma exacerbations are associated with the detection of viruses in respiratory secretions, with human rhinoviruses being the most common.^1^, ^2^ Thus, URTIs are a significant cause of morbidity and healthcare burden within the asthma population and there is an unmet need for therapeutics which prevent such infections from triggering exacerbations.

The mechanisms by which URTIs trigger exacerbations are poorly understood. One hypothesis is that asthmatic patients have impaired innate anti‐viral immunity. Several studies have reported evidence of delayed or deficient type I and/or type III interferon (IFN) response to virus infection in cells from asthmatic patients compared to healthy controls.^3^, ^4^ Wark *et al* showed impaired IFN‐β responses in rhinovirus‐infected asthmatic bronchial epithelial cells were associated with increased rhinovirus (RV) replication, which returned to normal levels after addition of exogenous IFN‐β.^5^ However, many other reports have failed to demonstrate this IFN deficiency (as reviewed by Edwards *et al* ^3^). More recently, IFN impairment has been observed in a subgroup of patients with severe, therapy‐resistant atopic asthma but not in patients with well‐controlled asthma.^6^, ^7^

The above findings led to the hypothesis that exogenous IFN‐β could be an effective treatment for the prevention of exacerbations triggered by URTI. Recombinant IFN‐β1a was evaluated in a previous study as an inhaled, on‐demand therapy for the prevention of asthma worsening following cold or flu symptoms, in a randomized, placebo‐controlled trial (NCT01126177^8^;). Although the primary endpoint, change in 6‐item asthma control questionnaire (ACQ‐6) score, was not met in the whole cohort, a planned subgroup analysis showed significant benefit of IFN‐β1a in patients with severe, difficult‐to‐treat asthma, both on the primary endpoint and lung function, in particular morning peak expiratory flow (mPEF).^8^

To confirm the results of the previous positive findings in the severe asthma subgroup, we performed a randomized, placebo‐controlled trial of on‐demand inhaled IFN‐β1a (hereafter AZD9412) in severe asthmatics. We hypothesized that inhaled AZD9412 would reduce the rate of virally triggered severe exacerbations and hence selected this to be the primary endpoint.

Some of the results of this trial have been previously reported in the form of an abstract.^9^

## 2 METHODS

Further detail on methods can be found in Supporting Information.

### 2.1 Study design

**Figure 1:**
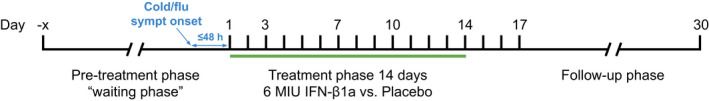
Study design

This was a randomized, double‐blind, placebo‐controlled trial evaluating the effect of inhaled AZD9412 on severe exacerbations upon URTI (Figure 1). Asthma patients (GINA steps 4‐5^10^), with a 24‐month history of ≥2 severe exacerbations related to URTI (1 within the last 12 months), were screened and recruited into a pre‐treatment waiting phase, during which they continued their previous treatment regimen (maintenance treatment with medium‐to‐high dose ICS [>250 μg fluticasone total daily dose] and a second controller medication). Patients were equipped with a home spirometer and a smartphone for questionnaires. During the pre‐treatment phase, while waiting for URTI symptoms to occur, a range of baseline assessments was completed, including lung function, blood, sputum, nasal lavage and urine samples for biomarkers, ECG recordings, ACQ‐6 and Asthma Quality of Life Questionnaire (AQLQ). On 4 consecutive days every month, patients were asked to complete a questionnaire with 10 questions on symptoms of colds and flu, in order to determine baseline. Patients were asked daily via an eDiary device if they thought they were developing a URTI (common cold or influenza). When a patient first reported onset of relevant symptoms (≥2 of sore throat, nasal symptoms [runny and/or blocked nose] different than normal, feeling feverish), they were randomized, as soon as possible but no later than 48 hours after the onset of symptoms, to a 14‐day course of 6 million units of once‐daily nebulized (iNeb, Philips) AZD9412 or placebo (both Rentschler Biopharma). From the onset of symptoms, patients rated their severity according to the modified Jackson cold score questionnaire on the eDiary each morning during the treatment and follow‐up periods, to clinically verify colds.^11^, ^12^ The following common cold/flu symptoms were included: sore throat, runny nose, sneeze, nasal congestion (blocked or stuffy nose), malaise (tiredness), fever (feverish/chills), headache, hoarseness, earaches and cough. Each symptom was rated on a scale from 0 = no symptoms, 1 = mild, 2 = moderate, 3 = severe.

Primary endpoint was the occurrence of severe exacerbations following onset of URTI for 14 days from start of treatment, compared with placebo. Secondary endpoints included occurrence of severe or moderate exacerbations (defined in Supporting Information) for up to 30 days from start of treatment, and changes from baseline in the 6‐item asthma control questionnaire (ACQ‐6),^13^ forced expiratory volume in 1 second (FEV_1_), PEF, reliever medication use, Asthma Symptom Diary scores, safety and tolerability. CompEx (**Comp**osite endpoint for **Ex**acerbations)^14^ was also evaluated. CompEx is a novel composite endpoint that captures clinically relevant asthma worsening episodes, based on a combination of asthma worsening diary events (morning and evening peak expiratory flow, symptoms and rescue medication use) plus severe exacerbation events. Patients attended the clinic every 3‐4 days during treatment and at days 17 and 30, for clinical examination and collection of blood and expectorated sputum.

A pre‐planned, un‐blinded, administrative interim analysis of the primary efficacy outcome was conducted when 50% of patients had completed the treatment phase, by sponsor personnel who were not involved in the conduct of the study. This was intended to facilitate investment decisions by the study sponsor. Blinded interim monitoring of the severe exacerbation event rate was also performed during the study to calculate, based on the number of events occurring in the two treatment arms combined, whether or not the sample size should be re‐estimated.

### 2.2 Exploratory biomarkers

The presence of 21 pathogens in nasal swab and sputum from the first 7 days of treatment was determined using the Respiratory Pathogens 21 qPCR kit (Fast Track Diagnostics). A patient was considered ‘virus‐positive’ when positive for any virus in any sample and time point.

Serum CXC‐motif chemokine ligand 10 (CXCL10), interleukin (IL)‐4, IL‐5, IL‐8, IL‐13, IL‐18, IFN‐gamma, tumour necrosis factor (TNF)‐α, TNF‐related apoptosis inducing ligand (TRAIL) and vascular endothelial growth factor A (VEGF‐A) concentrations were determined using V‐plex or U‐plex immunoassays (Mesoscale Discovery). Serum eosinophil‐derived neurotoxin (EDN) concentrations were measured by ELISA (MBL).

Sputum mRNA expression of CXCL10, GBP1, Mx1, OAS1 and IFIT2 was quantified by qRT‐PCR. Expression was normalized to the geometric mean of 3 housekeeping genes (PUM1, ACTB and HPRT1).

### 2.3 Statistical methods

The primary endpoint was analysed using a log‐binomial regression model with treatment arm and geographical region included as factors. Change from baseline in ACQ‐6 and in average area under the curve (AUC) of mPEF, asthma symptom score and reliever medication, was analysed using ANCOVA, with treatment arm and region as factors and the baseline assessment as covariate. The model for mPEF was also adjusted for sex, smoking and height. CompEx was analysed based on proportions of patients with events using a log‐binomial regression model as per the primary endpoint.

All efficacy analyses were carried out in the intention‐to‐treat (ITT) analysis set. Primary and key secondary endpoints were also evaluated in pre‐defined (clinically defined cold, northern and southern hemisphere, cold season [defined as September–December in the northern hemisphere and March–June in the southern hemisphere], virus positives) and *post hoc*‐defined subgroups (based on serum biomarker levels). Treatment arm differences in mRNA expression results, expressed as AUC change from baseline over the treatment period, were tested in linear models adjusted for baseline.

Thirty per cent of the patients in the placebo arm were expected to experience a severe exacerbation during treatment, based on previous studies in a similar patient population. N = 97 evaluable patients in each arm were required to provide 80% power to discover a relative risk reduction of 55% between the AZD9412 and placebo treatment arms at a significance level of 5%. Based on these calculations, 220 patients were scheduled to be randomized.

## 3 RESULTS

### 3.1 Patients

**Figure 2:**
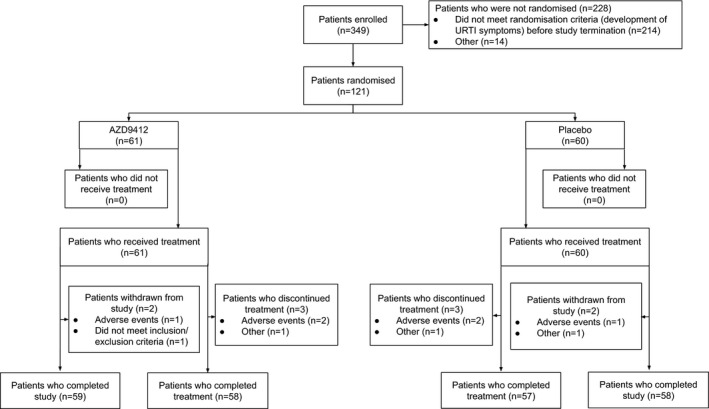
Patient disposition

Three‐hundred and forty‐nine patients were enrolled in the study, of whom 121 were randomized (ITT population). Patients were recruited in the following countries: Argentina (n = 48), United Kingdom (n = 31), South Korea (n = 21), Spain (n = 8), Australia (n = 6), France (n = 4) and Colombia (n = 3). Sixty‐one and 60 patients received AZD9412 and placebo, respectively (Figure 2). However, the trial was stopped following the pre‐planned interim analysis due to an unexpectedly low exacerbation rate and corresponding lack of evidence for differential response on the primary endpoint. The resulting reduction in patient numbers substantially reduced the statistical power of the study analyses. Demographics and patient characteristics at baseline are described in Table 1. There were no major differences between the active and placebo arms. Of the 228 patients not randomized, 214 did not meet the main randomization criteria (development of URTI symptoms) before study termination and 14 withdrew for other reasons (Figure 2). For those 121 patients who were randomized, the median waiting time from screening to randomization was 42 days (range 8‐224 days). Of the 121 randomized patients, 117 completed the study. Greater than 80% adherence to the study medication (ie 12 or more of the 14 once‐daily doses were administered) was achieved in 93.4% and 96.7% of the active and placebo arms, respectively.

**Table 1:** Demographics and patient characteristics (intention‐to‐treat (ITT) analysis set)

	AZD9412 (N = 61)	Placebo (N = 60)	Total (N = 121)
Age in years, mean (SD)	47.8 (13.0)	47.7 (14.1)	47.7 (13.5)
Sex, n (%)			
Male	13 (21.3)	17 (28.3)	30 (24.8)
Female	48 (78.7)	43 (71.7)	91 (75.2)
Race, n (%)			
White	41 (68.3)	44 (74.6)	85 (71.4)
Asian	12 (20.0)	12 (20.3)	24 (20.2)
Other	7 (11.7)	3 (5.1)	10 (8.4)
BMI, kg/m^2^, mean (SD)	29 (7.1)	29 (5.5)	29 (6.3)
Smoking status, n (%)			
Never	39 (63.9)	40 (66.7)	79 (65.3)
Current	1 (1.6)	3 (5.0)	4 (3.3)
Former	21 (34.4)	17 (28.3)	38 (31.4)
Lung function at screening			
n	58	56	114
pre‐BD FEV_1_ in L mean (SD)	2.2 (0.69)	2.2 (0.81)	2.2 (0.75)
FVC in L, mean (SD)	3.0 (0.89)	3.1 (1.00)	3.1 (0.94)
Number of severe exacerbations in the last 24 mo, n (%)			
1	1 (1.6)	0	1 (0.8)
2	19 (31.1)	24 (40.0)	43 (35.5)
3	22 (36.1)	18 (30.0)	40 (33.1)
4	9 (14.8)	11 (18.3)	20 (16.5)
≥5	10 (16.4)	7 (11.7)	17 (14.0)
Time since first symptoms of asthma^a^ in years, median (range)	21 (1.3, 55.0)	20.5 (1.8, 58.0)	21.0 (1.3, 58.0)
Time since last acute respiratory infection^a^ in months, median (range)	5.6 (0.9, 17.1)	6.4 (1.0, 12.6)	6.1 (0.9, 17.1)
Inhaled corticosteroid			
Medium dose	30 (49.2)	29 (48.3)	59 (48.8)
High dose	19 (31.1)	22 (36.7)	41 (33.9)
Equivalence dose level not possible to establish	12 (19.7)	9 (15.0)	21 (17.4)
LABA	59 (96.7)	59 (98.3)	118 (97.5)
LTRA	18 (29.5)	17 (28.3)	35 (28.9)
Theophylline	7 (11.5)	3 (5.0)	10 (8.3)
Oral corticosteroids	8 (13.1)	9 (15.0)	17 (14.0)

Abbreviations: BD, bronchodilator; BMI, body mass index; FEV_1_, forced expiratory volume in 1 s; FVC, forced volume capacity; kg, kilograms; L, litre; LABA, long‐acting β_2_‐agonist; LTRA, leukotriene receptor agonist; m^2^, square metre; N, number; SD, standard deviation.

^a^ At screening.

### 3.2 Exacerbations and CompEx in AZD9412 vs placebo

The number of patients with a severe exacerbation between days 1 and 14 was 7 (11.5%) and 5 (8.3%) in the active and placebo arm, respectively, with a rate ratio of 1.29 (95% CI 0.43 to 3.85, *p* = .645; Table 2). The proportion of patients with a severe exacerbation between days 1 and 30 was also similar between the two arms, as was time to severe exacerbation (data not shown).

**Table 2:** Proportion of patients with severe exacerbations

	AZD9412 (N = 61)	Placebo (N = 60)	Ratio of proportions (95% CI) AZD9412 vs Placebo	*p*‐value
	n (%)	n (%)		
Severe exacerbations Days 1 to 14	7 (11.5%)	5 (8.3%)	1.29 (0.43, 3.85)	.64
Severe exacerbations Days 1 to 30	8 (13.1%)	6 (10.0%)	1.25 (0.46, 3.41)	.66

Abbreviations: CI, confidence interval; N, number; vs, versus.

Similar to the findings on exacerbations, no statistically significant difference was found in the proportion of patients with a CompEx event between the active and placebo arms, although the reduced number of patients limited statistical power (Table 3).

**Table 3:** Proportion of patients with CompEx events

	AZD9412 (N = 61)	Placebo (N = 60)	Ratio of proportions (95% CI) AZD9412 vs Placebo	*p*‐value
	n (%)	n (%)		
Treatment Days 1 to 15	17 (27.9%)	18 (30.0)	0.85 (0.48, 1.52)	.59
Post‐treatment Days 15 to 30	9 (14.8)	12 (20.0)	0.74 (0.34, 1.61)	.45

Note

CompEx: composite endpoint for severe exacerbations; n: number; and vs: versus.

### 3.3 Lung function in AZD9412 versus placebo

**Figure 3:**
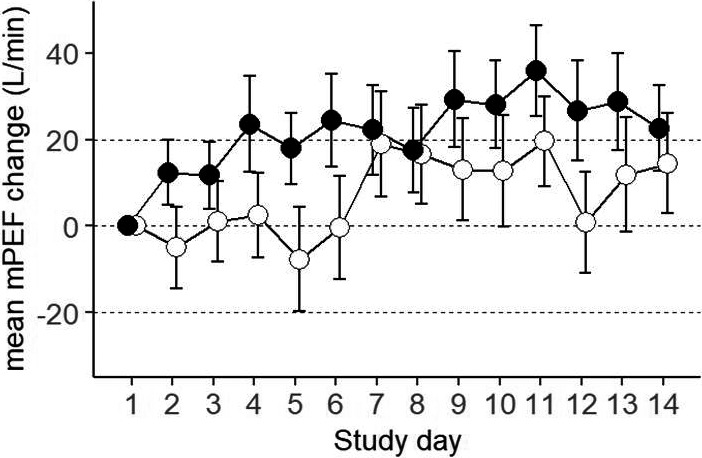
Mean mPEF AUC change from treatment baseline (day 1). Error bars are SEM. Closed circles: AZD9412; open circles: placebo

There was a tendency towards an increase in mean mPEF in both arms over the treatment period (Figure 3). The average AUC mPEF was statistically significantly greater in the active arm over days 1 to 7 compared with that seen in the placebo arm, LS mean of 21.9 L/min vs 2.1 L/min, with a LS mean difference of 19.7 L/min (95% CI 4.8 to 34.6, *p* = .01; Table 4). Likewise, there was an increase in average AUC mPEF in the active arm over days 1 to 14 compared with a decrease in the placebo arm [9.6 L/min vs –7.6 L/min, LS mean difference of 17.2 L/min (95% CI –0.5 to 34.9, *p* = .06; Table 4)]. There was no significant difference in mean percentage change from baseline FEV_1_ at any time point (Table S1).

**Table 4:** Morning peak expiratory flow (mPEF), area under the curve (AUC) for change from treatment baseline

Study period	Arm	N	LS mean (SE)	LS mean difference (95% CI)	*p*‐value
Days 1 to 7	AZD9412	56	21.9 (9.8)	19.7 (4.8, 34.6)	.01
	Placebo	59	2.1 (9.2)		
Days 1 to 14	AZD9412	55	9.6 (14.1)	17.2 (−0.5, 34.9)	.06
	Placebo	57	−7.6 (14.0)		

Abbreviations: CI, confidence interval; LS, least square; N, number; SE, standard error.

### 3.4 ACQ‐6, asthma symptom scores and reliever medication use

**Figure 4:**
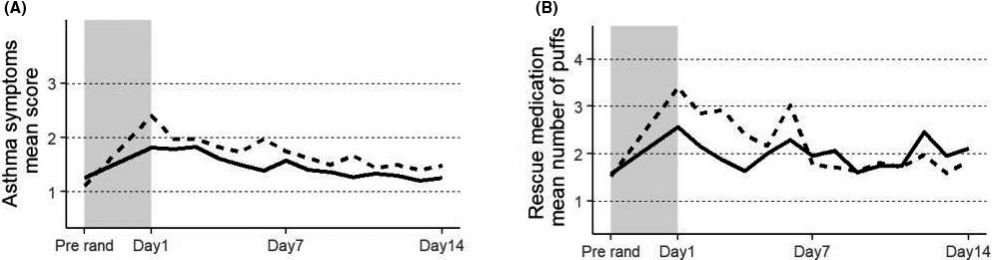
A, Asthma symptom score mean, at screening and during days 1‐7 of treatment. B, Reliever medication use, mean number of puffs, at screening and during days 1‐7 of treatment. Solid line: AZD9412; dashed line: placebo. Shaded area indicates the pre‐treatment waiting phase (from screening to randomization). Pre‐rand = pre‐randomization

There were no significant differences in ACQ‐6, asthma symptom scores or reliever medication use between AZD9412 and placebo (Figure S1, Tables S2 ‐ S4). The highest levels for asthma symptom scores and reliever medication use, from randomization to follow‐up, were observed at randomization, with a steady decline throughout the treatment period (Figure 4).

### 3.5 Pharmacodynamic effect of AZD9412

**Figure 5:**
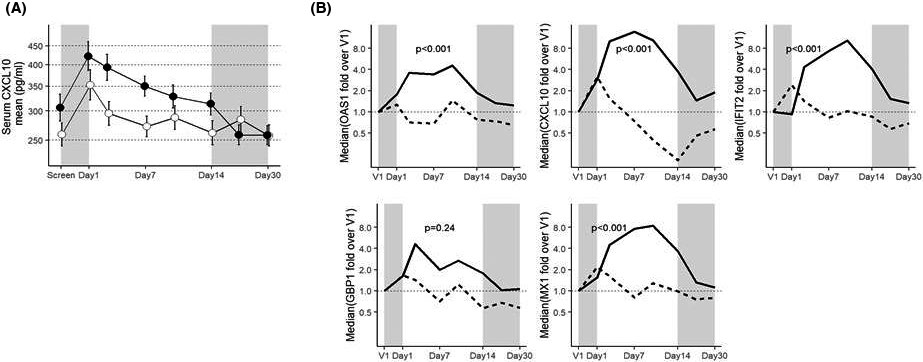
Pharmacodynamic effect of AZD9412 based on IFN‐response biomarkers. A, Mean serum CXCL10 concentrations throughout the study time course. Error bars are SEM. Closed circles: AZD9412; open circles: placebo. B, Median mRNA levels of sputum IFN‐stimulated genes throughout the treatment and follow‐up period. For each gene, mRNA levels are normalized to housekeeping genes (PUM1, ACTB and HPRT) and expressed as fold change over day 1 (V1). Solid line: AZD9412; dashed line: placebo. Shaded areas indicate the pre‐treatment waiting phase from screening to randomization (left area), and follow‐up period (right area)

Compared to screening, serum concentrations of the IFN‐response biomarker, CXCL10, increased in both placebo and AZD9412 arms at day 1, when patients had reported URTI symptoms, immediately before the first dose (Figure 5A). During the treatment period, patients on AZD9412 maintained significantly higher CXCL10 concentrations compared to placebo (*p* = .016, Figure 5A). By 3 days after end of treatment (visit 7), CXCL10 in the AZD9412 arm had returned to screening levels.

In sputum, the time courses of mRNA expression for 5 interferon‐stimulated genes (ISG) (CXCL10, Mx1, OAS1, IFIT2 and GBP1) were similar to serum CXCL10 protein, with a modest increase around day 1 in both arms compared to screening (Figure 5B). Throughout the treatment period, levels of all 5 ISG mRNA were higher in the AZD9412 arm compared to placebo, and this was statistically significant for all but GBP1 (*p* < .001). Unlike serum CXCL10 protein, in the AZD9412 arm all 5 ISG mRNA increased further from day 1 to day 2 and peaked later (around day 3 for GBP1 and day 7‐10 for the others).

### 3.6 Pre‐defined subgroup analyses

Sub‐division of the cohort based on northern or southern hemisphere or clinically verified colds (92% of the ITT population) showed similar results to the main ITT population (Tables S5 and S6). Patients were divided into serum CXCL10‐low, medium and high subgroups based on pre‐treatment baseline levels, and the results were similar between all 3 subgroups (Table S7A‐C).

Overall, 52% of evaluable patients were virus‐positive, of whom 31% were rhinovirus‐positive (Table S8). There were no significant differences in virus positivity between the AZD9412 and placebo arms. Like the ITT population, the virus‐positive subgroup showed a statistically significant effect of AZD9412 on mPEF (days 1‐7, LS mean difference 25.6 L/min (1.98, 49.2), *p* = .03) (Table S9A and Table 4). However, as in the ITT population, there was no effect of AZD9412 on any other secondary endpoint (Table S9A). In the virus‐negative subgroup, the difference in mPEF between AZD9412 and placebo was not statistically significant [days 1‐7, LS mean difference 20.0 L/min (−3.82, 43.8), *p* = .10; Table S9B].

### 3.7 Additional post hoc subgroup analyses

Subgroup analyses not pre‐defined in the protocol should be regarded as exploratory, and any findings would need to be validated in future studies.

To investigate the time course of inflammatory events before, during and after a URTI, and to determine whether AZD9412 has an impact on these, we conducted an exploratory analysis of 9 biomarkers in serum: IL‐4, IL‐5, IL‐8, IL‐13, IL‐18, IFN‐gamma, TNF‐alpha, TRAIL and VEGF‐A.

To identify biomarkers which were altered by AZD9412 treatment, mean relative difference between AZD9412 and placebo in change from baseline at end of treatment was calculated. IL‐18 was significantly increased in the AZD9412 arm [mean relative difference 0.87 (0.77, 0.98), *p* = .02; Table S10]).

**Figure 6:**
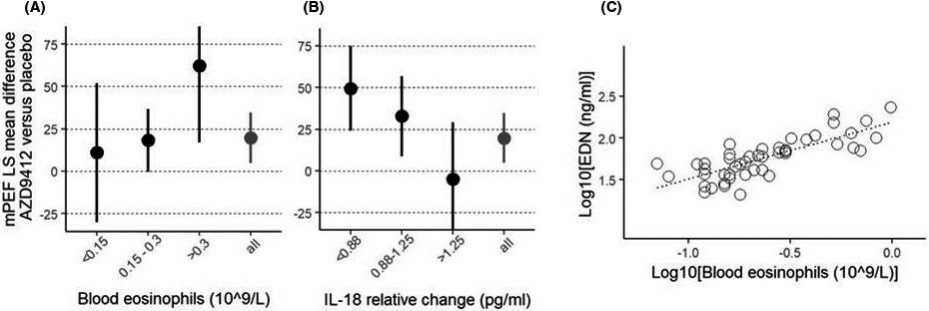
A, Mean mPEF AUC change (day 1‐7) in patients sub‐divided on blood eosinophil counts at screening. Patient numbers in low, mid and high subgroups were 9, 32 and 12 for AZD9412 and 19, 27 and 12 for placebo. B, Mean mPEF AUC change (day 1‐7) in patients sub‐divided on mean IL‐18 relative change from screening to treatment baseline. Patient numbers in low, mid and high subgroups were n = 14, 29 and 13 for AZD9412, and n = 14, 30 and 13 for placebo. A‐B, All = main ITT population. Error bars are 95% confidence intervals. C, Scatterplot of blood eosinophil counts versus EDN (log_10_‐scale) based on all patients with both EDN and eosinophil count data at screening (n = 47, excluding one patient whose blood eosinophil count = 0 and 3 patients with missing eosinophil count data). Correlation between EDN and blood eosinophil counts (log_10_‐scale) is 0.78 (Pearson's correlation coefficient)

Nineteen biomarkers and clinical variables were explored as potentially predictive of treatment effect on mPEF, 4 of which indicated an interaction with treatment (*p* < .1): blood eosinophils (visit 1 or 2), blood neutrophils (visit 1 or 2), serum IL‐18 and serum TRAIL relative change at pre‐treatment baseline (visit 2/visit 1). Further analyses of these, based on biomarker quartile level, showed an increase in mPEF treatment response across subgroups of low, mid and high eosinophil levels, while there was a decreasing trend in mPEF treatment response across subgroups of low, mid and high IL‐18 levels (Table 5 and Figure 6A‐B).

**Table 5:** Morning peak expiratory flow (mPEF), area under the curve (AUC) change from baseline in subgroups defined by four different biomarkers: serum IL‐18 fold change, serum TRAIL fold change, eosinophils (10^9^/L) at baseline and neutrophils (10^9^/L) at baseline

Biomarker (Q1,Q3)	Treatment period	mPEF difference AZD9412 – Placebo, (*p*‐value)		
		Low group marker < Q1	Mid group Q1 ≤ marker ≤Q3	High group marker > Q3
IL18 FCH (0.88,1.25)	Days 1 to 7	49.5 (0.0007)	32.8 (0.008)	−4.9 (0.76)
	Days 1 to 14	38.9 (0.02)	28.0 (0.054)	−7.22 (0.73)
EOSINOPHILS (0.15, 0.33)	Days 1 to 7	−0.06 (0.99)	21.1 (0.04)	56.1 (0.005)
	Days 1 to 14	−2.1 (0.94)	27.7 (0.014)	53.2 (0.021)
NEUTROPHILS (3.4,5.84)	Days 1 to 7	38.5 (0.15)	18.6 (0.09)	5.1 (0.71)
	Days 1 to 14	13.8 (0.62)	14.3 (0.23)	14.5 (0.41)
TRAIL FCH (0.81, 1.36)	Days 1 to 7	−19.5(0.21)	30.9 (0.009)	9.3 (0.58)
	Days 1 to 14	−26.3 (0.14)	25.4 (0.073)	20.0 (0.35)

Note

These biomarkers were selected from a total of 19 biomarkers, as those which showed an indication of being predictive of morning PEF, AUC change from baseline in a multiple regression model.

Abbreviations: FCH, fold change; IL‐, interleukin; L, litre; Q, quartile; TRAIL, TNF‐related apoptosis inducing ligand.

We also aimed to determine whether a soluble biomarker could be utilized to identify patients with high blood eosinophils in this cohort. Eosinophil‐derived neurotoxin (EDN) is a granule protein released from eosinophils upon activation and as such, has been described in the literature as a robust biomarker of activated eosinophils.^15^ We therefore measured EDN concentrations in serum from 51 patients at screening. Serum EDN concentrations correlated strongly with baseline blood eosinophil counts (*r* ^2^ = .61; Figure 6C).

### 3.8 Safety and Tolerability

The safety profile of AZD9412 was consistent with previous clinical experience,^9^ and no new safety concerns were identified. The proportion of patients reporting adverse events (AEs) was 47.5% in the AZD9412 arm compared with 33.3% in the placebo arm (Table S11). Most AEs were mild or moderate in intensity. Three patients reported serious AEs (SAEs), which were all asthma exacerbations resulting in hospitalization and occurring in the AZD9412 arm. All SAEs were assessed as causally unrelated to the investigational product by the Investigator. There were no notable findings in safety laboratory variables, vital signs, ECG or liver function tests.

## 4 DISCUSSION

Using a trial designed to achieve therapeutic dosing following URTI symptoms, we have evaluated the efficacy of on‐demand AZD9412 on the rate of severe asthma exacerbations. We confirmed pharmacodynamic response to AZD9412, showing increased IFN‐response biomarkers in serum and sputum. However, the reduction from the planned 220 to 121 randomized patients and the unexpectedly low exacerbation rate substantially reduced the power of the study to detect a significant benefit of AZD9412 during the 14 days of treatment compared with placebo. While the primary endpoint (rate of severe exacerbations) and most secondary endpoints were not met, there was a statistically significant improvement of AZD9412 on mPEF during the first 7 days of treatment that was similar to the previous trial of inhaled IFN‐β1a in patients with asthma.^8^ The difference of 19.7 L/min, however, is of limited clinical significance. Our *post hoc* exploratory analyses indicated a greater mPEF improvement in patients with either high blood eosinophils at screening or low serum IL‐18 response at the time of URTI symptoms. Finally, asthma worsenings were generally mild and tended to peak at the start of treatment.

We originally aimed to randomize 220 patients based on an expected event rate of 30% in the placebo arm. However, following a pre‐planned interim analysis after 50% of patients had been randomized, a low event rate was observed (8% in placebo; see Table 2), with no difference between AZD9412 and placebo. These findings led to the decision to terminate the study following the randomization of 121 patients. However, it was assessed before termination of the trial that the sample size was large enough to have sufficient power to evaluate key secondary endpoints (ACQ‐6 and mPEF). We acknowledge that there were limitations which may explain our over‐estimation of the anticipated event rates. Although we used previous clinical experience and epidemiological data to estimate expected event rates,^16^, ^17^, ^18^ most previous exacerbation studies had longer observation periods than this trial. Furthermore, although there are data on how many exacerbations are linked to URTI,^1^, ^2^ it is less clear what proportion of URTI leads to asthma exacerbations. Since only one course of treatment was given per subject, we relied on reasonable odds that a single URTI would lead to an exacerbation.

As a pre‐specified exploratory endpoint, we utilized CompEx, a recently described composite measure of asthma events^14^ which has shown increased sensitivity for the detection of treatment efficacy on asthma exacerbations. In line with previous findings, the total number of CompEx events observed in our trial (17 in AZD9412; 18 in placebo) was approximately 2.5‐ to 3‐fold greater than the number of severe exacerbations. However, as for the primary endpoint, no differences between AZD9412 and placebo were found for CompEx events.

We demonstrated a clear pharmacodynamic effect using IFN‐response biomarkers in this study. Upon activation of its receptor, IFNAR, IFN‐β triggers a transcriptional response giving rise to the induction of hundreds of ISG.^19^ CXCL10 is one such ISG, whose product is a secreted chemokine and thus serves as a robust soluble protein biomarker of IFN‐β activity (both endogenous IFN‐β and AZD9412). As expected, serum CXCL10 and sputum ISG mRNA were increased when patients reported URTI symptoms. However, while in the placebo arm these biomarkers steadily returned to baseline levels within the first week of treatment, they remained upregulated in the AZD9412 arm during treatment and declined to baseline levels thereafter. These findings are similar to those shown in a previous trial of inhaled IFN‐β1a in asthmatics.^8^ The increase of the IFN‐response biomarkers upon URTI symptoms suggests that these were virus‐related events, even though only 52% of randomized patients tested virus‐positive. The reasons for the discrepancy between the rates of virus positivity (52%) and clinically confirmed colds (92%) using the criteria of Jackson *et al* or Predy *et al* ^11^, ^12^ are unknown, but highlight the need to develop sensitive diagnostic tests for viral URTI.

The previous trial of inhaled IFN‐β1a had a similar on‐demand design, and Djukanovic *et al* reported that IFN‐β1a prevented an increase in ACQ‐6 in a subgroup of patients with difficult‐to‐treat asthma (British Thoracic Society steps 4‐5) reporting URTI symptoms.^8^ This was associated with a significant improvement in mPEF in the IFN‐β1a arm versus placebo over the treatment period. Although our trial replicates these mPEF findings, we saw no effect of AZD9412 on ACQ‐6. The previous trial did not report on the rate of exacerbations. Despite the difference in the ACQ‐6 outcome, our study population was comparable with the difficult‐to‐treat subgroup in Djukanovic *et al*.^8^ We used the GINA steps 4‐5 classification rather than those of the British Thoracic Society. Although the criteria are similar, there may be subtle differences between them,^10^, ^20^ and a documented history of severe exacerbations related to URTI was required for enrolment. It is notable that in our trial, ACQ‐6 values were higher at screening and, unlike in the previous trial, did not increase in the placebo arm following URTI symptoms (see Figure S1).

In a *post hoc* exploratory analysis, we observed a trend towards an improved mPEF response in two subgroups: patients with high blood eosinophils and patients with a low IL‐18 relative change at the time of URTI symptoms. Eosinophils have previously been linked to impaired anti‐viral responses and viral exacerbations.^21^, ^22^, ^23^ IL‐18, a key IL‐1‐related cytokine and a component of the inflammasome, has been reported to be protective against viral infections,^24^ and most notably was inversely associated with lower respiratory tract symptom worsenings in asthmatics experimentally challenged with RV16.^25^ IL‐18 is known to activate natural killer (NK) cells and to modulate innate‐lymphoid cells,^26^ cell types that have been implicated in anti‐viral immunity and virus‐triggered exacerbations. Thus, it is tempting to speculate that asthmatics with high blood eosinophils or low IL‐18 may have impaired innate anti‐viral immunity and would therefore be more likely to benefit from IFN‐β1a therapy. However, this hypothesis would require confirmation.

Our trial investigated therapeutic dosing of AZD9412 which, as opposed to prophylactic dosing, can be taken ‘as needed’, minimizing patient burden. On the other hand, prophylactic dosing of AZD9412 would prime the airway prior to infection and thus prevent the initial establishment of infection. Indeed, in vitro investigations have shown more profound anti‐viral activity when cells are pre‐treated with IFN‐β as compared to treatment at the time of infection.^5^ Furthermore, a recent study by Watson et al demonstrated that pre‐treatment of cultured airway epithelial cells with exogenous IFN‐β provided an anti‐viral effect on influenza infection which lasted for several days.^27^ One potential limitation of our trial design in terms of mimicking rapid, on‐demand treatment is the unavoidable lag time between the subject reporting URTI symptoms and attending the clinic for randomization. However, our randomization criteria required that the first dose be taken within 48 hours of first reporting of URTI symptoms. Moreover, most patients started treatment within 24 hours of URTI symptoms. Our approach to initiation of treatment was endorsed by the US FDA prior to commencing the study. The FDA recognized the difficulties associated with using viral testing to identify suitable patients for initiation and accepted patient reporting of relevant symptoms as a proxy for identification of patients with a viral URTI. Thus, we believe we succeeded in starting AZD9412 treatment as early as possible in a clinical trial setting. Another possible limitation is that daily questioning about symptoms may have led to over‐reporting of URTI‐like symptoms (eg by patients with hay fever or rhinosinusitis). This could explain the low virus positivity and low rate of exacerbations.

Therapeutic dosing of AZD9412 may simply be too late to prevent the virus from triggering a worsening of asthma. Consistent with this notion, we showed in the placebo arm that both reliever medication use and asthma symptom scores had already peaked at the time of randomization and started to improve thereafter. This is consistent with the findings of a similar clinical trial for the rhinovirus capsid binder vapendavir.^28^ Furthermore, in a study of asthmatics experimentally challenged with RV16, the peak of both upper and lower respiratory tract symptoms occurred simultaneously, around 4 days post‐inoculation.^29^ These findings would suggest that earlier intervention with AZD9412 may be required to prevent virus‐triggered events in asthma, for example seasonal prophylaxis, post‐exposure prophylaxis or through identifying a more sensitive predictor of an oncoming URTI.^30^

Finally, it is possible that most patients could mount a sufficiently robust endogenous IFN response (ie were not type I IFN deficient). However, our subgroup analysis showed no evidence of increased efficacy in patients with low CXCL10 levels at pre‐treatment baseline, suggesting that the extent of the endogenous IFN response had no bearing on efficacy. Furthermore, we saw no evidence of increased exacerbation rates in the CXCL10‐low subgroup (data not shown). As viral load was not quantified in this study, and serum CXCL10 levels had already peaked at pre‐treatment baseline, it is not clear to what extent variation in baseline CXCL10 levels are due to differences in the efficiency of the IFN response or simply due to differences in viral load.

In summary, respiratory viral infections did not have the expected impact and, due to the low exacerbation rate, our evaluation of on‐demand inhaled AZD9412 versus placebo for the prevention of severe asthma exacerbations following URTI symptoms was stopped early. AZD9412 showed no differential effect on severe exacerbations, but did give rise to an improvement in mPEF, a response which in an exploratory analysis tended to be greater in patients with either high blood eosinophils or low serum IL‐18 relative change at pre‐treatment baseline. The finding that changes in asthma endpoints were minimal, and had already peaked at randomization, suggests that early detection of asthma worsening, and identifying patients more likely to deteriorate due to respiratory viruses, is essential for optimal efficacy of on‐demand IFN‐β1a therapy. Our findings should be taken into consideration for the future development of inhaled IFN‐β1a.

## 5 ETHICS

This study was performed in accordance with the ethical principles that have their origin in the Declaration of Helsinki and that are consistent with International Council for Harmonisation (ICH)/Good Clinical Practice (GCP) and applicable regulatory requirements and the AstraZeneca policy on Bioethics and Human Biological Samples. Each participating centre's Institutional Review Board (IRB) or an Ethics Committee (EC) approved the final version of the Clinical Study Protocol (CSP), including the final version of the Informed Consent Form (ICF). In the United Kingdom, approval was from NHS Health Research Authority NRES Committee South Central—Hampshire B, REC reference 15/SC/0256. Patients provided signed and dated consent forms prior to randomization.

## CONFLICTS OF INTEREST

CM, MO, PG, AM, MA, MF, CAD, AC, JP, KK and CW are employees and shareholders of AstraZeneca. MK is a former employee of AstraZeneca. PM and RM are directors and shareholders in Synairgen plc. TH reports personal fees and non‐financial support from AstraZeneca and GlaxoSmithKline, and personal fees from Chiesi and Vectura, outside of the submitted work.

## AUTHOR CONTRIBUTIONS

CM, MO, PG, AM, MA, MF, CAD, PM, RM and TH contributed to the design and execution of the study and the interpretation of the results. MO performed the statistical analyses, with contributions from CM and PG CM, AC, JP, KK, MK and CW performed the biomarker analyses. CM wrote the manuscript, with substantial contributions from MO, PG, AM, CAD, MF and TH. All co‐authors critically reviewed the manuscript.

## Supporting information

Supplementary Material

## Data Availability

Data underlying the findings described in this manuscript may be obtained in accordance with AstraZeneca's data sharing policy described at https://astrazenecagrouptrials.pharmacm.com/ST/Submission/Disclosure.
