# Supplementary material for: INEXAS: A Phase 2 Randomized Trial of On‐demand Inhaled Interferon Beta‐1a in Severe Asthmatics
Source: Clin Exp Allergy. 2020 Nov 3;51(2):273–83. doi: 10.1111/cea.13765 (PMC7984268; doi:10.1111/cea.13765)

**INEXAS: A Phase 2 Randomised Trial of On-Demand Inhaled Interferon Beta-1a in Severe Asthmatics**

Christopher McCrae, Marita Olsson, Per Gustafson, Anna Malmgren, Malin Aurell, Malin Fagerås , Carla A. Da Silva, Anders Cavallin, Jonathan Paraskos, Karin Karlsson, Cecilia Wingren, Phillip Monk, Richard Marsden, Tim Harrison.

**Online Data Supplement**

**Supplementary Methods**

**Full clinical study design**

This was a randomised, double-blind, placebo-controlled, multi-centre, parallel group, Phase IIa study in adult asthma patients receiving maintenance treatment with medium to high dose inhaled corticosteroids (ICS; >250 μg fluticasone dry powder formulation equivalents total daily dose, as defined in GINA 2014, and a second controller medication (e.g. long-acting β_2_-agonists [LABA]), to investigate whether treatment with inhaled AZD9412, initiated after the onset of symptoms of a URTI, could prevent severe exacerbations.

Eligible patients had a documented history of at least 2 severe asthma exacerbations within the last 24 months, of which at least 1 had occurred during the last 12 months. These exacerbations were suspected by the patient to have been triggered by an upper respiratory tract infection (i.e. related to symptoms of a common cold or the flu).

The study consisted of a pre-treatment phase followed by a 14-day treatment phase. Patients were screened for entry into the pre-treatment phase and remained in this phase until they developed symptoms of a common cold or the flu. During this pre-treatment phase, patients were asked daily (via an electronic Patient Reported Outcome [ePRO] device) whether they thought they had a common cold or the flu. In addition, patients were provided with a home spirometer and during 4 consecutive days every month they were asked to complete an asthma symptom questionnaire, including rescue medication use and PEF measurements, using the ePRO device. When the patient answered “yes” to indicate the onset of a common cold or flu, a subquestionnaire where patients answered questions on presence and severity of cold/flu symptoms opened up in the ePRO device, and arrangements were made to evaluate the patient at the study site to ascertain their eligibility. If eligible they entered the treatment phase.

Baseline assessments were performed and patients were randomised 1:1 to receive 6 MIU (24 μg; metered dose) inhaled AZD9412 or placebo once daily for 14 days (delivered by the I-neb® device [Philips Respironics]). Treatment started as soon as possible but no later than 48 hours after the onset of the first symptoms of a common cold or the flu. Patients were assessed with regards to exacerbations and changes in respiratory symptoms and reliever medication use at home twice daily using an ePRO device. Lung function was measured both at home by the patients and at the clinic. Patients administered the study drug under the supervision of study staff on clinic visit days and by themselves at home on non-visit days.

Patients were encouraged to contact the study staff in case of questions related to dosing or handling of the inhalation device. There were 5 clinic visits during the treatment phase. There were 2 visits after the end of treatment; efficacy and safety were monitored until 2 to 3 weeks after end of treatment when a final follow-up visit was performed.

**Flow chart of study design**

**Primary outcome:**

To evaluate the efficacy of AZD9412 compared to placebo, after 14 days of treatment, in preventing severe exacerbations following onset of an upper respiratory infection (URTI) in asthmatic patients on top of their regular background treatment.

A severe exacerbation was defined as worsening of asthma symptoms and at least one of the following:

a) Use of systemic corticosteroids (or a temporary increase in a stable oral corticosteroid background dose) for at least three days.

b) An unscheduled visit or emergency room visit due to asthma symptoms that requires at least one dose of systemic corticosteroids.

c) An inpatient hospitalisation due to asthma requiring at least one dose of systemic corticosteroids

**Secondary outcomes:**

1. To evaluate the preventive or attenuating effect of AZD9412 compared to placebo on deterioration of secondary efficacy endpoints during and after URTI. Analysed variables include:

a) Proportion of patients with severe exacerbations in active group, with onset during day 1-30 after start of treatment

b) Proportion of patients with moderate exacerbations* in active group

c) Time to exacerbation (moderate and severe)

d) Patient reported outcomes

(i) Asthma Control Questionnaire (ACQ-6) as determined by change from baseline

(ii) Asthma Symptom Score as determined by area under the curve (AUC) over day 1-14 as change from the baseline level.

(iii) Asthma Quality of life questionnaire (AQLQ)

e) Reliever medication use as determined by AUC over day 1-14 as change from the baseline level

f) Lung function i.e. peak expiratory flow (PEF), FEV_1_

(i) PEF and FEV_1_ measured daily at home as determined by AUC over day 1-14 as change from the baseline level.

(ii) FEV_1_ measured at the clinic at each visit.

The effect on the outcome variables were analysed separately:

(i) During day (D)1-D7 and D1-D14 for event variables and during D1-D7, D8-D14 and D15-D30 (from start of treatment) for other variables

(ii) for different modified intention to treat populations, e.g.

1. virus positive in nose/sputum,

II. patients reaching a certain cold severity score (e.g. using the Jackson score level)

*Definition of moderate exacerbation: “A temporary increase in controller therapy in order to prevent a severe event supported by a sustained (≥ 2 days) worsening in at least one key control metric i.e. asthma score, reliever medication use, night time awakening or morning PEF”

2. To assess the safety and tolerability of AZD9412 compared to placebo on:

a. Adverse events (AE);

b. supine pulse and blood pressure;

c. standard haematology;

d. biochemistry and urinalysis;

e. C-reactive protein (CRP);

f. 12-lead electrocardiogram (ECG);

g. physical examination;

h. lung function [spirometry (peak expiratory flow (PEF), forced expiratory volume in 1 second (FEV_1_), forced vital capacity (FVC)), diffusing capacity of the lung for carbon monoxide (DLCO)].

**Overview of assessments and monitoring:**

**Screening visit (Visit 1)**

Patients were included into the Pre-treatment phase of the study according to the “Subject inclusion and exclusion criteria” (see below) and were assessed according to the Assessment Schedule (below).

| **Assessment schedule** | | | | | | | | | |
| --- | --- | --- | --- | --- | --- | --- | --- | --- | --- |
|  | **Pre-treatment phase** | **Treatment phase** | | | | | **Follow-up** | |  |
| **Visit** | **1** | **2** | **3** | **4** | **5** | **6** | **7** | **8** |  |
| **Day** | **Enrolment** | **1** | **3/4** | **7±1** | **10±1** | **14/15 (End of treatment)** | **17±1** | **30-35 post randomisation** | **Unscheduled^l^** |
| Informed consent | X |  |  |  |  |  |  |  |  |
| Informed consent for optional PGx | X^a^ | X^a^ |  |  |  |  |  |  |  |
| Allocate E-code | X |  |  |  |  |  |  |  |  |
| Demography | X |  |  |  |  |  |  |  |  |
| Medical and surgical history | X | X |  |  |  |  |  |  |  |
| Asthma/exacerbation history confirmation | X^b^ |  |  |  |  |  |  |  |  |
| Smoking history/habits | X | X |  |  |  |  |  |  |  |
| Height and weight | X | X^c^ |  |  |  |  | X^c^ | X^c^ |  |
| Physical examination | X | X | X^d^ | X^d^ | X^d^ | X^d^ | X^d^ | X |  |
| Digital and paper 12- lead ECG (dECG and pECG) | X^e^ | X^f^ |  |  |  | X^g^ |  | X^e^ |  |
| Vital Signs (BP and pulse) | X | X^h^ | X^h^ | X^h^ | X^h^ | X^h^ | X | X |  |
| Inclusion/Exclusion criteria | X | X |  |  |  |  |  |  |  |
| Clinical chemistry, haematology, urinalysis | X^i^ | X^i^ | X^j^ | X^j^ | X^j^ | X^i^ | X^j^ | X^i^ |  |
| HIV, hepatitis B and C tests | X |  |  |  |  |  |  |  |  |
| Urine pregnancy test, dip stick  (females of childbearing potential) | X | X |  |  |  |  | X | X |  |
| LH and FSH (females) | X^k^ |  |  |  |  |  |  |  |  |
| AEs and SAEs^m^ | X | X | X | X | X | X | X | X | X |
| Concomitant medication | X | X | X | X | X | X | X | X | X |
| Reversibility test/pre- and post-bronchodilator spirometry | X^n^ |  |  |  |  |  |  |  |  |
| Spirometry | X^o^ | X^p^ | X^q^ | X^q^ | X^q^ | X^q^ | X | X |  |
| Diffusing capacity of the lung for carbon monoxide (DLCO)^r^ | X |  |  |  |  |  | X |  |  |
| Assessment of asthma exacerbation |  | X | X | X | X | X | X | X | X |
| Blood sampling for biomarker analysis | X | X^s^ | X^s^ | X^s^ | X^s^ | X^s^ | X | X |  |
| Expectorate sputum sampling for biomarker analysis | X | X^s^ | X^s^ | X^s^ | X^s^ | X^s^ | X | X |  |
| Urine for biomarker analysis | X | X^s^ |  |  |  |  |  |  |  |
| Nasal lavage for biomarker analysis | X | X^s^ |  |  |  |  |  |  |  |
| I-neb^®^ device demonstration | X |  |  |  |  |  |  |  |  |
| I-neb^®^ device training |  | X |  |  |  |  |  |  |  |
| Training for home monitoring equipment; hand‑held spirometer and ePRO device | X | X |  |  |  |  |  |  |  |
| Dispense (D)/collect (C) home monitoring equipment | D |  |  |  |  |  |  | C |  |
| Randomisation |  | X |  |  |  |  |  |  |  |
| Asthma Control Questionnaire (ACQ-6) | X | X |  | X |  | X |  |  |  |
| Asthma Quality of Life Questionnaire (AQLQ[S]) | X | X |  |  |  | X |  |  |  |
| Asthma Daily Diary | X^t^ | X |  |  |  |  |  | X |  |
| Modified Jackson Cold symptoms questionnaire | X^u^ | X |  |  |  |  |  | X |  |
| Optional blood sample for PGx |  | X^v^ |  |  |  |  |  |  |  |
| Dispense (D)/collect (C) I-neb^®^ device |  | D |  |  |  | C |  |  |  |
| IP accountability (D=Dispense, R=Return) |  | D | D |  |  | R |  |  |  |
| Administration of IP at the study site^x^ |  | X | X | X | X | X |  |  |  |
| Administration of IP at home (between visits)^x^ |  |  |  |  |  |  |  |  |  |
| Patient inhalation technique training (InSight) |  | X | X^y^ | X^y^ | X^y^ |  |  |  |  |
| Patient compliance check (InSight) |  |  | X | X^z^ | X^z^ |  |  |  |  |
| Compliance of IP check |  |  | X | X | X | X |  |  |  |
| Home PEF, FEV_1_, eDiary adherence |  | X | X | X | X | X | X | X |  |
| Collect/download home monitoring information |  | X | X | X | X | X | X | X |  |

a May be signed at either Visit 1 or Visit 2

b Including number of severe exacerbations within the last 24 months, and number of severe exacerbations triggered by a cold or flu within the last 12 months

c Weight only

d Brief physical examination (lung and heart auscultation)

e At Visits 1 and 8 print-outs from triplicate dECGs will be evaluated by the Investigator (if needed via the Central ECG Laboratory on request base)

f At Visit 2 (randomisation) triplicate dECG will be taken pre-dose (analyzed by the Central ECG Laboratory)

g At Visit 6 triplicate dECG will be taken (analyzed by the Central ECG Laboratory)

h BP and pulse will be performed pre-dose and 15 minutes post-dose at each treatment visit after randomisation (from Visit 2 to Visit 6) when the IP has been administered at the study site

i Full safety lab panel

j Reduced safety lab panel

k FSH and LH for women under the age of 50 considered to be post-menopausal to determine that the hormones are in the post-menopausal range and that the women thereby not need any contraceptives

l Unscheduled visits may be initiated as needed, and additional assessments performed at these visits, at the discretion of the investigator

m SAEs are collected from informed consent throughout the study. AEs are collected from randomisation (Visit 2) throughout the treatment period and including the follow-up period (Visits 7 and 8)

n If historical post-bronchodilator reversibility or methacholine or histamine challenge information is not available a reversibility test can be performed. If reversibility testing fails during Visit 1, a methacholine challenge may be performed during Visit 1

o If not performed as part of the reversibility test

p At Visit 2 spirometry will be done pre-dose and 30 minutes post-dose

q At Visits 3-6 spirometry will be done pre-dose

r DLCO will be done at selected study sites and for selected patients only

s Samples for biomarker analysis will be taken pre-dose

t During the Pre-Treatment Phase, starting the day following Visit 1, patients will complete the Asthma Daily Diary each morning and evening for four consecutive days once every month. In case a common cold/flu is confirmed with the patient experiencing at least 2 of the following symptoms (a sore throat, nasal symptoms (runny and/or blocked nose) different than normal, feels feverish), they will also start completing the Asthma Daily Diary

u Starting the day following Visit 1, patients will complete the modified Jackson Cold symptoms questionnaire each morning for seven consecutive days.

From the eighth day, following Visit 1, recording of the level of cold symptoms will start when the patients have answered ‘yes’ to the cold/flu question

v Optional blood sample for genetic research may be obtained at any time during the study after obtaining exploratory genetic consent. The genetic blood sample should ideally be drawn during the randomisation visit and through the same cannula used to draw blood samples for the main study

x The IP should be taken once daily preferably in the morning, at approximately the same time of day. There must be a gap of at least 12 hours between doses

y Optional at Visits 3, 4 and 5

z Optional at Visits 4 and 5

**Pre-treatment phase (Between Visit 1 and Visit 2)**

During the Pre-treatment phase the patient was asked daily (via smartphone) if they believed they had a cold. When the patient experienced symptoms, contact with the patient was established and he/she was asked to come to the clinic for further evaluation and potential randomisation/start of treatment as soon as possible, but within 48h of symptom onset. The patient could also contact the clinic spontaneously upon symptoms.

During the Pre-treatment phase patients filled out the “Daily Asthma Diary” (asthma symptoms and PEF morning and evening) at home for three days during the first month and then for one day once every month.

**Start of treatment phase, randomisation visit (Visit 2)**

The patient was evaluated at the clinic for potential inclusion in the Treatment phase in order to receive first dose of treatment as soon as possible but within 48h of onset of first symptom of URTI according to the “Randomisation inclusion and exclusion criteria” (see below). If judged eligible for inclusion into the Treatment phase, assessments were performed according to the Assessment schedule. If the patient was judged not eligible for inclusion into the Treatment phase he/she re-entered the Pre-treatment phase.

**Treatment and follow-up phase (Visit 2 to Visit 9)**

The patient was randomised (Day 1) to a 14-day treatment of AZD9412 once daily or Placebo once daily, in a 1:1 ratio. First dose had to be administered within 48 hours of onset of first symptom of URTI. The Treatment Phase consisted of 7 clinic visits on Days 1, 3-4, 6-8, 9-11, 14, 16-18, and 30-35. Patients were contacted daily by phone on non-clinical visit days during the Treatment phase (from Visit 2 to Visit 7) to confirm drug administration, verify that home monitoring had been performed, ask active questions about exacerbation symptoms, treatment and health care contacts, and collect AE information.

Doses were delivered through once daily inhalation using a nebuliser device. Subjects were given training prior to using the device and took their first dose of study medication under the supervision of study staff. Subjects were assessed to ensure that they could use the device competently prior to home dosing, and were thus deemed competent to take their doses at home. On clinic visit days, subjects were encouraged to take their doses in the clinic so that any further training needed could be identified and provided. Ideally, the study medication was to be taken at approximately the same time of day, with a minimum gap of 8 hours between doses. Throughout the study, safety information was obtained including concomitant medication use and AE.

**Subject Inclusion and Exclusion Criteria:**

Number of Subjects:

- Pre-treatment phase: approximately 400
- Randomised to treatment: 121 (61 to AZD9412, 60 to placebo)
- Evaluable patients: 121

Study inclusion criteria (for entry into Pre-treatment phase):

For inclusion in the Pre-treatment phase patients must fulfil all of the following criteria:

1. Male or female aged 18 to 75 years (inclusive) of age at the time of screening.

2. History of physician-diagnosed asthma requiring treatment with medium-to-high dose inhaled corticosteroids (ICS) (>250 μg fluticasone dry powder formulation equivalents total daily dose, as defined in GINA 2014; see Appendix Chapter 5) and a LABA. The medium or high dose ICS plus LABA can be any combination inhaler or 2 separate inhalers. Patients must have taken the ICS ≥fluticasone 250 μg or the equivalent daily plus LABA for at least 12 months prior to the date the informed consent is obtained, with or without another controller such as oral corticosteroids (OCS), theophylline, tiotropium, or leukotriene receptor antagonists

3. Documented post-bronchodilator (post-BD) reversibility in FEV_1_ of >12% and >200 mL in FEV_1_ within 12 months prior to Visit 1. If historical documentation is not available, reversibility must be demonstrated and documented at Visit 1

4. Must answer “Yes” to the question “Does a cold make your asthma worse?”

5. To have had at least two documented severe asthma exacerbations within the last 24 months, of which at least one has occurred during the last 12 months, and based on information provided to the Investigator by the patient it is suspected that these exacerbations were related to URTI.

6. Patient consents to the investigator taking full control of their asthma management.

7. Females of childbearing potential must be non-pregnant (verified by urine dipstick pregnancy test) and using a medically acceptable adequate form of birth control and agree to maintain this usage throughout the study (i.e. during both Pre-treatment and Treatment phases).

8. Motivation (in the Investigator’s opinion) to complete all study visits, the ability to communicate well with the Investigator and be capable of understanding the nature of the research and its treatment including its risks and benefits

9. For inclusion in the optional genetic research (blood sampling for genetic testing), patients must provide a separate signed and dated informed consent. (If a patient declines to participate in the optional exploratory genetic research there will be no penalty or loss of benefit to the patient. The patient will not be excluded from other aspects of the study.)

**Study exclusion criteria (for entry into Pre-treatment phase)**

A patient must not be entered into the study if they meet any of the following criteria:

1. Any condition, including findings in the medical history or in the pre-study assessments that in the opinion of the Investigator, constitutes a risk or a contraindication for the participation of the patient in the study or that could interfere with the study objectives, conduct or evaluation.

2. Lung disease other than asthma (i.e. chronic obstructive pulmonary disease, cystic fibrosis).

3. Patients who are taking >10mg/day of oral corticosteroids for treatment of their asthma.

4. Current participation in another clinical trial or participation in a clinical trial where the patient has received a dose of a test product (IMP) within 12 weeks prior to entry into the study for small molecules and within 6 months prior to entry into the study for biologicals.

5. Patients who currently have, or have had within the past 3 months, any significant underlying medical condition(s) that could impact interpretation of results e.g. infections, haematological disease, malignancy, renal, hepatic, coronary heart disease or other cardiovascular disease, including arrhythmias, endocrinological or gastrointestinal disease.

6. History of hypersensitivity to AZD9412 or to any of the drug preparation excipients.

7. Significant history of depressive disorder or suicidal ideation. Specifically; individuals with current severe depression (i.e. a low mood, which pervades all aspects of life and an inability to experience pleasure in activities that formerly were enjoyed); individuals with a past history of depression that required hospitalisation or referral to psychiatric services in the past 5 years; individuals who currently feel suicidal or have attempted suicide in the past.

8. History of epilepsy or seizures after the age of 5 years, other than febrile childhood seizure(s).

9. History of drug or alcohol abuse within 12 months prior to screening.

10. Patients who have hepatic serum enzyme levels ≥ 2.5 times the normal range.

11. Female who is breast-feeding, pregnant (verified by urine dipstick pregnancy test) or intends to become pregnant.

12. Patients who are unable to demonstrate an acceptable nebuliser technique and spirometry techniques.

13. Patients that have previously been included in studies evaluating AZD9412.

**Randomisation inclusion criteria (for entry into Treatment phase)**

For inclusion into the Treatment phase patients must fulfil all of the following criteria:

1. The patient thinks that he/she has a cold or influenza [as opposed to e.g. allergic rhinitis (especially important to try to differentiate from rhinitis symptoms during e.g. pollen season)]

2. The patient experiences at least 2 of the following symptoms indicating a URTI for no longer than 48h:

a) A sore or scratchy throat

b) Nasal symptoms (runny and / or blocked nose), different than normal

c) Feels feverish and/or muscle pain

3. Persisting infection symptoms, but no longer than 48 hours from onset to administration of first dose

**Randomisation exclusion criteria (for entry into Treatment phase)**

A patient must not be randomised (entered into the Treatment phase) if they meet any of the following criteria:

1. The patient has already met the criterion for a severe exacerbation and/or started treatment for a bacterial infection, e.g. any of the below:

a) The patient has already started taking, or has increased their oral steroids for an asthma exacerbation; and/or

b) The patient has already started taking antibiotics for the treatment of an exacerbation; and/or

c) The patient has been seeking health care (hospital or other physician contact) due to worsening of asthma symptoms.

2.Since the Screening Visit, the patient has taken or started to take any medication other than their asthma medications or has been diagnosed with any further (lung) disease, that the Investigator deems as not suitable for inclusion into the Treatment Phase.

3. The patient is breast feeding or pregnant (verified by urine dipstick pregnancy test).

**Rationale for inclusion/exclusion criteria**

Criteria listed are needed for ensuring a proper diagnosis of asthma and common cold, for ensuring patients’ safety, for ensuring patient’s ability to adhere to study treatment and monitoring, or for excluding conditions that might bias the efficacy assessment.

**Statistical Analysis**

The efficacy analysis was made on the intention to treat (ITT) analysis set. The ITT analysis set is defined as randomised patients who received at least one dose of the study medication.

Analysis of the primary endpoint was addressed using a log-binomial regression model. Treatment was included as factor in the models as well as the hemisphere (northern/southern) and presence of cold season (defined as September-December in the Northern Hemisphere and March-June in the Southern Hemisphere). The back-transformed estimated treatment effect (i.e., the ratio of proportions, AZD9412 over placebo, was presented with 2-sided 95% confidence intervals and p-values (two-sided alternatives). The model was estimated using the PROC GENMOD data step in SAS® with binomial and log specified for distribution and link, respectively.

The presence of hemisphere-treatment interactions was explored graphically and by a Breslow-Day’s test (two-sided with a significance level of 10%) using PROC FREQ in SAS®.

**Analyses of secondary endpoints**

ACQ-6 was analyzed as change from baseline after first week of treatment and after completed treatment. Asthma symptom score, reliever medication use, and morning PEF (mPEF) were analyzed as average AUC change from baseline (days 1-7 and 1-14), by means of an ANCOVA model with treatment group and region as factors and the baseline assessment as covariate. The model used for mPEF was in addition adjusted for sex, smoking status (never, former, current) and height. Patients with less than 50% daily registrations during treatment period were excluded from the analyses of AUC-based variables.

The percentage change from baseline in clinic visit FEV_1_ was analyzed using a repeated measures mixed model with treatment group, visit, interaction between treatment and visit, region, sex and smoking status as fixed effects, and baseline FEV_1_ and height as covariates.

**Exploratory analyses**

The treatment group difference in mRNA expression of five interferon-stimulated genes (CXC motif chemokine ligand 10 (CXCL10), myxoma resistance protein 1 (Mx1), oligoadenylate synthetase 1 (OAS1), interferon inducible transcript 2 (IFIT2) and guanylate binding protein 1 (GBP1) was tested in baseline adjusted linear models where the response variable was defined as AUC change from baseline (day of randomisation) over the 14-day treatment period.

Nine biomarkers measured in serum (interleukin (IL)-4, -5, -8, -13, -18, IFN-gamma, tumor necrosis factor (TNF)-alpha, TNF-related apoptosis inducing ligand (TRAIL) and vascular-endothelial growth factor A (VEGF-A) were investigated for treatment group difference in change from baseline. For five of them (IL-4, -5, -13, IFNg, and TNFa) more than 50% of the subjects had missing data for Day 1 (baseline) and/or for Day 14 (end of treatment) and were therefore excluded from further analyses. Each of the remaining four biomarkers were analyzed on log-scale in a linear model with end of treatment measurement as response, treatment group as factor and baseline as covariate.

An exploratory investigation to identify potential predictive biomarkers was performed using mPEF AUC change from baseline as response variable. A total of 19 different biomarkers were tested: ACQ-6 at screening and at Day 1, cold score at Day 1, virus positive at Day 1, number of days (0,1 or 2) between first symptoms and Day 1, relative change Day 1/screening in CXCL10, in IL-8, in IL-18, in VEGF-A, and in TRAIL, mRNA expression at Day 1 for OAS1, for GBP1, for CXCL10, for MX1, and for IFIT2, blood eosinophils at screening and at Day 1, blood neutrophils at screening and at Day 1. For each biomarker an ANCOVA model was set up with treatment group and the biomarker included as main effects and as an interaction. The model included in addition mPEF baseline, sex, height, smoking status, and region. Those biomarkers for which the interaction term showed an indication of significance (p-value < 0.1) were further investigated in subgroup analyses. Three subgroups were defined based on the biomarker quartiles; a low group (biomarker < Q1), a mid-group (Q1 < biomarker < Q3), and a high group (biomarker >Q3). The same mPEF ANCOVA model, except for exclusion of the biomarker terms, was then fitted within each subgroup to check for an increasing or decreasing treatment effect pattern across the subgroups.

Correlation between serum EDN and blood eosinophils at screening was calculated on logged data using Pearson correlation coefficient.

**Rationale for sample size**

Based on observed exacerbation rates in previous studies with a similar patient population as in this study, the proportion of patients experiencing a virus-induced severe exacerbation was estimated to be 30% in the placebo group. Assuming a true placebo proportion of 30% and with 97 evaluable patients in each treatment group, a two-sided Chi-square test at a significance level of 5% for the difference in proportion between the active and placebo groups, would discover a relative risk reduction (RRR) of 55% with a power of 80%. The sample size was calculated using nQuery, version 7.0. Based on these calculations, a target of 220 randomised patients was set to ensure at least 194 evaluable patients.

However, colds did not have the impact on asthma patients that was expected and, due to the low exacerbation rate, the trial was stopped early, following a pre-planned interim analysis. There was no effect of AZD9412 on due to the unexpectedly low exacerbation rate. Asthma worsenings were generally mild and tended to peak at randomisation, possibly contributing to the lack of benefit of AZD9412 on other asthma endpoints.

**Supplementary Tables**

| **Table E1.** **FEV_1_, percentage change from baseline treatment comparisons, MMRM analysis (ITT analysis set)** | | | | | | |
| --- | --- | --- | --- | --- | --- | --- |
|  |  | **Percent change from baseline** | | **Comparison with placebo** | | |
| Time point | Treatment group | n | LS Mean (SE) | LS Mean difference | 95% CI | p-value |
| Visit 3 | AZD9412 (N=61) | 60 | 4.73 (3.38) | -1.31 | (-6.86, 4.24) | 0.640 |
|  | Placebo (N=60) | 60 | 6.04 (3.24) |  |  |  |
| Visit 4 | AZD9412 (N=61) | 60 | 4.97 (3.49) | 0.78 | (-5.35, 6.91) | 0.801 |
|  | Placebo (N=60) | 58 | 4.19 (3.38) |  |  |  |
| Visit 5 | AZD9412 (N=61) | 59 | 4.12 (3.44) | -1.20 | (-7.08, 4.69) | 0.688 |
|  | Placebo (N=60) | 56 | 5.32 (3.33) |  |  |  |
| Visit 6 | AZD9412 (N=61) | 59 | 3.23 (3.43) | -1.98 | (-7.83, 3.87) | 0.504 |
|  | Placebo (N=60) | 57 | 5.21 (3.32) |  |  |  |
| Visit 7 | AZD9412 (N=61) | 61 | 4.89 (3.68) | -2.51 | (-9.50, 4.49) | 0.479 |
|  | Placebo (N=60) | 58 | 7.40 (3.60) |  |  |  |
| Visit 8 | AZD9412 (N=61) | 61 | 6.54 (3.93) | 0.61 | (-7.35, 8.57) | 0.880 |
|  | Placebo (N=60) | 59 | 5.93 (3.84) |  |  |  |

Estimate of the mean percentage change from baseline at each week in the AZD9412 group was compared with the placebo group using a repeated measures analysis.

The model was: percentage change in FEV_1_ = treatment group + visit + region + gender + smoking status + baseline FEV_1_ + height + treatment-by-visit interaction using an unstructured variance-covariance matrix. Baseline was the last non-missing assessment prior to randomisation.

Abbreviations:

CI confidence interval; FEV_1_ forced expiratory volume in 1 second; ITT intention to treat; LS least square; MMRM mixed-effect model repeated measures; N number of patients in treatment group; n number of patients included in analysis; SE standard error.

Data source: Table 11.2.9.2.2.

**Table E2. Total asthma symptom score (night time + day time), AUC for change
from baseline**

| **Study period** | **Arm** | **N** | **LS Mean (SE)** | **LS Mean Difference (95%CI)** | **P-value** |
| --- | --- | --- | --- | --- | --- |
| Days 1 to 7 | AZD9412 | 55 | -0.09 (0.1) | 0.07 (-0.16,0.31) | 0.53 |
|  | Placebo | 55 | -0.16 (0.1) |  |  |
| Days 1 to14 | AZD9412 | 53 | -0.31 (0.14) | 0.02 (-0.29, 0.33) | 0.89 |
|  | Placebo | 50 | -0.33 (0.14) |  |  |

Abbreviations:

AUC: area under the curve; n: number; LS: least square; SE: standard error; CI: confidence interval

**Table E3. Reliever medication use (night time + day time), AUC for change from baseline in number of puffs.**

| **Study period** | **Arm** | **N** | **LS Mean (SE)** | **LS Mean Difference (95%CI)** | **P-value** |
| --- | --- | --- | --- | --- | --- |
| Days 1 to 7 | AZD9412* | 54 | -0.42 (0.24) | -0.35 (-0.93,0.24) | 0.24 |
|  | Placebo | 55 | -0.07 (0.25) |  |  |
| Days 1- 14 | AZD9412* | 52 | -0.58 (0.27) | -0.06 (-0.68, 0.55) | 0.84 |
|  | Placebo | 50 | -0.52 (0.28) |  |  |

*Extreme subject excluded due to having a mean number of puffs which was 10 standard deviations above the daily mean of all other patients.

Abbreviations:

AUC: area under the curve; n: number; LS: least square; SE: standard error; CI: confidence interval

**Table E4. Asthma control questionnaire (ACQ)-6, change from baseline**

| **Day of treatment** | **Arm** | **N** | **LS Mean (SE)** | **LS Mean Difference (95%CI)** | **P-value** |
| --- | --- | --- | --- | --- | --- |
| Day 7 | AZD9412 | 50 | 0.03 (0.11) | 0.14 (-0.13,0.41) | 0.30 |
|  | Placebo | 51 | -0.12 (0.12) |  |  |
| Day 14 | AZD9412 | 56 | -0.15 (0.14) | 0.04 (-0.29, 0.37) | 0.79 |
|  | Placebo | 53 | -0.19 (0.14) |  |  |

Abbreviations:

n: number; LS: least square; SE: standard error; CI: confidence interval

**Table E5. Proportion of patients with severe exacerbations by hemisphere.**

|  | AZD9412 (N=61) | Placebo (N=60) | Ratio of proportions (95%CI) AZD9412 vs Placebo | p-value |
| --- | --- | --- | --- | --- |
|  | n (%) | n (%) |  |  |
| Northern hemisphere  Days 1 to 14 | 5 of 31 (16.1%) | 3 of 33 (9.1%) | 1.83 (0.48, 7.00) | 0.38 |
| Southern hemisphere  Days 1 to 14 | 2 of 30 (6.7%) | 2 of 27 (7.4%) | 0.65 (0.11, 3.67) | 0.62 |

**Table E6. Key secondary endpoints in subjects with clinically confirmed colds.**

| Endpoint | Study period | Arm | N | LS Mean (SE) | LS Mean Difference (95%CI) | P-value |
| --- | --- | --- | --- | --- | --- | --- |
| ACQ6 change from baseline | Day 7 | AZD9412 | 49 | 0.06 (0.12) | 0.12 (-0.15, 0.39) | 0.38 |
|  |  | Placebo | 49 | -0.06 (0.12) |  |  |
|  | Day 14 | AZD9412 | 53 | -0.13 (0.15) | 0.10 (-0.24, 0.44) | 0.55 |
|  |  | Placebo | 50 | -0.23 (0.16) |  |  |
| Morning PEF AUC change from baseline | Days 1 to 7 | AZD9412 | 53 | 20.8 (10.38) | 21.2 (5.41, 36.91) | 0.01 |
|  |  | Placebo | 53 | -0.36 (9.64) |  |  |
|  | Days 1 to 14 | AZD9412 | 52 | 8.0 (14.93) | 17.6 (-1.36, 36.57) | 0.07 |
|  |  | Placebo | 51 | -9.6 (14.69) |  |  |
| Asthma symptom score AUC change from baseline | Days 1 to 7 | AZD9412 | 45 | -0.15 (0.17) | 0.09 (-0.20, 0.37) | 0.55 |
|  |  | Placebo | 46 | -0.23 (0.15) |  |  |
|  | Days 1 to 14 | AZD9412 | 44 | -0.12 (0.21) | 0.11 (-0.25, 0.48) | 0.53 |
|  |  | Placebo | 42 | -0.24 (0.20) |  |  |
|  |  | Placebo |  |  |  |  |

**Table E7A. Key secondary endpoints in CXCL10-low subjects. Subjects were classified as low if their baseline (day 1, prior of dosing) CXCL10 was below 251 pg/ml (Q1 of all subjects with CXCL10 measured at study day 1).**

| Endpoint | Study period | Arm | N | LS Mean (SE) | LS Mean Difference (95%CI) | P-value |
| --- | --- | --- | --- | --- | --- | --- |
| ACQ6 change from baseline | Day 7 | AZD9412 | 9 | -0.21 (0.26) | -0.10 (-0.78, 0.59) | 0.77 |
|  |  | Placebo | 17 | -0.12 (0.21) |  |  |
|  | Day 14 | AZD9412 | 8 | -0.60 (0.33) | -0.71 (-1.59, 0.17) | 0.11 |
|  |  | Placebo | 16 | 0.11 (0.23) |  |  |
| Morning PEF AUC change from baseline | Days 1 to 7 | AZD9412 | 10 | 33.4 (23.0) | 29.0 (-16.1, 74.1) | 0.19 |
|  |  | Placebo | 20 | 4.4 (16.2) |  |  |
|  | Days 1 to 14 | AZD9412 | 9 | 36.5 (30.3) | 31.9 (-18.8, 82.6) | 0.20 |
|  |  | Placebo | 19 | 4.6 (23.3) |  |  |
| Asthma symptom score AUC change from baseline | Days 1 to 7 | AZD9412 | 10 | -0.25 (0.24) | 0.11 (-0.50, 0.73) | 0.71 |
|  |  | Placebo | 19 | -0.37 (0.18) |  |  |
|  | Days 1 to 14 | AZD9412 | 10 | -0.42 (0.33) | 0.08 (-0.79, 0.94) | 0.86 |
|  |  | Placebo | 17 | -0.50 (0.27) |  |  |
| Reliever medication use AUC change from baseline | Days 1 to 7 | AZD9412* | 9 | -0.24 (0.66) | -0.63 (-2.3, 1.0) | 0.44 |
|  |  | Placebo | 19 | 0.39 (0.47) |  |  |
|  | Days 1 to 14 | AZD9412* | 9 | -0.10 (0.74) | 0.24 (-1.7, 2.1) | 0.80 |
|  |  | Placebo | 17 | -0.34 (0.59) |  |  |

*Extreme subject excluded

Abbreviations:

CXCL10: C-X-C motif chemokine ligand 10; pg/ml: picogram per millilitre; Q: quartile; n: number; LS: least square; SE: standard error; CI: confidence interval; ACQ: asthma control questionnaire; PEF: peak expiratory flow; AUC: area under the curve

**Table E7B. Key secondary endpoints in CXCL10-middle subjects. Subjects were classified as middle if their baseline (day 1, prior of dosing) CXCL10 was between 251 pg/ml and 553 pg/ml (between Q1 and Q3 of all subjects with CXCL10 measured at study day 1).**

| Endpoint | Study period | Arm | N | LS Mean (SE) | LS Mean Difference (95%CI) | P-value |
| --- | --- | --- | --- | --- | --- | --- |
| ACQ-6 change from baseline | Day 7 | AZD9412 | 26 | 0.09 (0.16) | 0.15 (-0.30, 0.61) | 0.49 |
|  |  | Placebo | 19 | -0.06 (0.18) |  |  |
|  | Day 14 | AZD9412 | 32 | 0.02 (0.17) | 0.05 (-0.46, 0.56) | 0.84 |
|  |  | Placebo | 22 | -0.03 (0.21) |  |  |
| Morning PEF AUC change from baseline | Days 1 to 7 | AZD9412 | 33 | 1.2 (13.5) | 18.6 (-1.2, 38.4) | 0.07 |
|  |  | Placebo | 25 | -17.4 (15.2) |  |  |
|  | Days 1 to 14 | AZD9412 | 33 | -7.7 (16.2) | 18.6 (-5.1, 42.2) | 0.12 |
|  |  | Placebo | 25 | -26.3 (18.2) |  |  |
| Asthma symptom score AUC change from baseline | Days 1 to 7 | AZD9412 | 31 | 0.03 (0.12) | 0.09 (-0.25, 0.42) | 0.61 |
|  |  | Placebo | 23 | -0.05 (0.13) |  |  |
|  | Days 1 to 14 | AZD9412 | 30 | -0.05 (0.15) | 0.09 (-0.34, 0.51) | 0.68 |
|  |  | Placebo | 22 | -0.13 (0.17) |  |  |
| Reliever medication use AUC change from baseline | Days 1 to 7 | AZD9412 | 31 | -0.52 (0.25) | -0.10 (-0.80, 0.61) | 0.79 |
|  |  | Placebo | 23 | -0.42 (0.28) |  |  |
|  | Days 1 to 14 | AZD9412 | 30 | -0.60 (0.28) | -0.20 (-1.0, 0.61) | 0.62 |
|  |  | Placebo | 22 | -0.40 (0.32) |  |  |

Abbreviations:

CXCL10: C-X-C motif chemokine ligand 10; pg/ml: picogram per millilitre; Q: quartile; n: number; LS: least square; SE: standard error; CI: confidence interval; ACQ: asthma control questionnaire; PEF: peak expiratory flow; AUC: area under the curve

**Table E7C. Key secondary endpoints in CXCL10-high subjects. Subjects were classified as high if their baseline (day 1, prior of dosing) CXCL10 was above 553 pg/ml (Q3 of all subjects with** **CXCL10 measured at study day 1).**

| Endpoint | Study period | Arm | N | LS Mean (SE) | LS Mean Difference (95%CI) | P-value |
| --- | --- | --- | --- | --- | --- | --- |
| ACQ-6 change from baseline | Day 7 | AZD9412 | 14 | 0.03 (0.22) | 0.13 (-0.45, 0.71) | 0.66 |
|  |  | Placebo | 15 | -0.10 (9.25) |  |  |
|  | Day 14 | AZD9412 | 15 | -0.35 (0.18) | -0.09 (-0.55, 0.37) | 0.70 |
|  |  | Placebo | 15 | -0.26 (0.20) |  |  |
| Morning PEF AUC change from baseline | Days 1 to 7 | AZD9412 | 12 | 67.4 (28.7) | 30.1 (-10.5, 70.8) | 0.13 |
|  |  | Placebo | 14 | 37.3 (25.6) |  |  |
|  | Days 1 to 14 | AZD9412 | 12 | 53.1 (30.0) | 24.7 (-25.2, 74.5) | 0.31 |
|  |  | Placebo | 13 | 28.5 (28.8) |  |  |
| Asthma symptom score AUC change from baseline | Days 1 to 7 | AZD9412 | 13 | -0.20 (0.21) | -0.09 (-0.63, 0.44) | 0.71 |
|  |  | Placebo | 13 | -0.11 (0.22) |  |  |
|  | Days 1 to 14 | AZD9412 | 12 | -0.69 (0.20) | -0.17 (-0.68, 0.34) | 0.49 |
|  |  | Placebo | 11 | -0.52 (0.23) |  |  |
| Reliever medication use AUC change from baseline | Days 1 to 7 | AZD9412 | 13 | -1.10 (0.43) | -0.78 (-1.90, 0.32) | 0.16 |
|  |  | Placebo | 13 | -0.31 (0.44) |  |  |
|  | Days 1 to 14 | AZD9412 | 12 | -1.37 (0.38) | -0.45 (-1.40, 0.50) | 0.33 |
|  |  | Placebo | 11 | -0.91 (0.43) |  |  |

Abbreviations:

CXCL10: C-X-C motif chemokine ligand 10; pg/ml: picogram per millilitre; Q: quartile; n: number; LS: least square; SE: standard error; CI: confidence interval; ACQ: asthma control questionnaire; PEF: peak expiratory flow; AUC: area under the curve

**Table E8. Virus positive subjects. Number of subjects with nasal lavage or sputum samples at visit 2, 3 and 4 were 89, 63, and 57 respectively, and in total 112 different subjects with at least one available result. Numbers shown are n (row %).**

|  | Visit 2  89 subjects | | Visit 3  63 subjects | | Visit 4  57 subjects | | Visit 2, 3 or 4  112 subjects | |
| --- | --- | --- | --- | --- | --- | --- | --- | --- |
|  | All virus | RV | All virus | RV | All virus | RV | All virus | RV |
| AZD9412 | 15 (34%) | 5 (11%) | 15 (54%) | 6 (21%) | 12 (46%) | 4 (15%) | 29 (51%) | 10 (18%) |
| Placebo | 14 (31%) | 0 (0%) | 15 (43%) | 6 (17%) | 13 (42%) | 5 (16%) | 29 (53%) | 8 (15%) |
| Total | 29 (33%) | 5 (5.6%) | 30 (48%) | 12 (19%) | 25 (44%) | 9 (21%) | 58 (52%) | 18 (16%) |

Abbreviations:

n: number; RV: rhinovirus

**Table E9A. Key secondary endpoints in virus positive subjects. Subjects were classified as virus positive if positive for any virus at visit 2, 3 or 4 (first week of study).**

| Endpoint | Study period | Arm | N | LS Mean (SE) | LS Mean Difference (95%CI) | P-value |
| --- | --- | --- | --- | --- | --- | --- |
| ACQ-6 change from baseline | Day 7 | AZD9412 | 23 | -0.078 (0.208) | -0.08 (-0.48, 0.33) | 0.71 |
|  |  | Placebo | 27 | -0.002 (0.182) |  |  |
|  | Day 14 | AZD9412 | 28 | 0.042 (0.243) | 0.03 (-0.43, 0.50) | 0.88 |
|  |  | Placebo | 28 | 0.009 (0.223) |  |  |
| Morning PEF AUC change from baseline | Days 1 to 7 | AZD9412 | 27 | 31.0 (16.5) | 25.6 (1.98, 49.2) | 0.03 |
|  |  | Placebo | 28 | 5.41 (14.5) |  |  |
|  | Days 1- 14 | AZD9412 | 27 | 23.2 (15.0) | 24.0 (-4.10, 52.2) | 0.09 |
|  |  | Placebo | 26 | -0.80 (14.0) |  |  |
| Asthma symptom score AUC change from baseline | Days 1 to 7 | AZD9412 | 27 | -0.21 (0.16) | -0.18 (-0.50, 0.14) | 0.27 |
|  |  | Placebo | 25 | -0.03 (0.15) |  |  |
|  | Days 1- 14 | AZD9412 | 26 | -0.23 (0.22) | -0.27 (-0.73, 0.20) | 0.26 |
|  |  | Placebo | 21 | 0.03 (0.21) |  |  |
| Reliever medication use AUC change from baseline | Days 1 to 7 | AZD9412* | 26 | -0.27 (0.35) | -0.097 (-0.78, 0.59) | 0.78 |
|  |  | Placebo | 25 | -0.18 (0.31) |  |  |
|  | Days 1- 14 | AZD9412* | 25 | 0.20 (0.37) | -0.018 (-0.78, 0.74) | 0.96 |
|  |  | Placebo | 21 | 0.22 (0.34) |  |  |

*Extreme subject excluded

Abbreviations:

n: number; LS: least square; SE: standard error; CI: confidence interval; ACQ: asthma control questionnaire; PEF: peak expiratory flow; AUC: area under the curve

**Table E9B. Key secondary endpoints in virus negative subjects**

| Endpoint | Study period | Arm | N | LS Mean (SE) | LS Mean Difference (95%CI) | P-value |
| --- | --- | --- | --- | --- | --- | --- |
| ACQ-6 change from baseline | Day 7 | AZD9412 | 24 | -0.000 (0.145) | 0.23 (-0.14, 0.60) | 0.23 |
|  |  | Placebo | 21 | -0.232 (0.165) |  |  |
|  | Day 14 | AZD9412 | 25 | -0.188 (0.203) | 0.07 (-0.43, 0.57) | 0.77 |
|  |  | Placebo | 21 | -0.259 (0.225) |  |  |
| Morning PEF AUC change from baseline | Days 1 to 7 | AZD9412 | 25 | 19.6 (17.4) | 20.0 (-3.82, 43.8) | 0.10 |
|  |  | Placebo | 26 | -0.37 (16.8) |  |  |
|  | Days 1- 14 | AZD9412 | 24 | 13.9 (19.0) | 13.5 (-12.7, 39.8) | 0.30 |
|  |  | Placebo | 26 | 0.36 (18.3) |  |  |
| Asthma symptom score AUC change from baseline | Days 1 to 7 | AZD9412 | 25 | -0.06 (0.13) | 0.21 (-0.13, 0.56) | 0.22 |
|  |  | Placebo | 25 | -0.27 (0.15) |  |  |
|  | Days 1- 14 | AZD9412 | 24 | -0.26 (0.16) | 0.27 (-0.13, 0.67) | 0.18 |
|  |  | Placebo | 25 | -0.53 (0.18) |  |  |
| Reliever medication use AUC change from baseline | Days 1 to 7 | AZD9412 | 25 | -0.99 (0.31) | -0.44 (-1.22, 0.34) | 0.27 |
|  |  | Placebo | 25 | -0.56 (0.34) |  |  |
|  | Days 1- 14 | AZD9412 | 24 | -1.08 (0.40) | -0.24 (-1.20, 0.72) | 0.61 |
|  |  | Placebo | 25 | -0.84 (0.43) |  |  |

Abbreviations:

n: number; LS: least square; SE: standard error; CI: confidence interval; ACQ: asthma control questionnaire; PEF: peak expiratory flow; AUC: area under the curve

**Table E10. Comparison of serum biomarkers in AZD9412 versus placebo groups. Biomarkers are analysed on log-scale. Tests of relative difference between treatment groups in change from baseline at End of Treatment.**

| Serum Biomarker | Arm | N | Mean relative Diff (95%CI) | P-value |
| --- | --- | --- | --- | --- |
| IL-8 | AZD9412 | 58 | 0.92 (0.71, 1.20) | 0.56 |
|  | Placebo | 55 |  |  |
| IL-18 | AZD9412 | 59 | 0.87 (0.77, 0.98) | 0.02 |
|  | Placebo | 55 |  |  |
| VEGF-A | AZD9412 | 59 | 0.94 (0.82, 1.08) | 0.40 |
|  | Placebo | 55 |  |  |
| TRAIL | AZD9412 | 59 | 0.93 (0.82, 1.08) | 0.23 |
|  | Placebo | 57 |  |  |

Abbreviations:

n: number; CI: confidence interval; IL-: interleukin; VEGF: vascular endothelial growth factor; TRAIL: TNF-related apoptosis inducing ligand

**Table E11. Adverse events**

| Adverse events in any category during double-blind phase - patient level (safety analysis set) | | |
| --- | --- | --- |
|  | **Number (%) of patients** | |
|  | **AZD9412**  **(N=61)** | **Placebo**  **(N=60)** |
| Any AE | 29 (47.5) | 20 (33.3) |
| Any AE with outcome = death | 0 | 0 |
| Any SAE (including events with outcome=death) | 3 (4.9) | 0 |
| Any AE leading to discontinuation of IP | 2 (3.3) | 3 (5.0) |
| Any other significant adverse event (OAE) | 0 | 0 |

Abbreviations:

N: number; AE: adverse event; SAE: serious adverse event; IP: investigational product

**Figure E1: ACQ-6 change at day 7 from pre-treatment baseline. Error bars are 95% confidence intervals. Statistical significance was assessed using ANCOVA.**


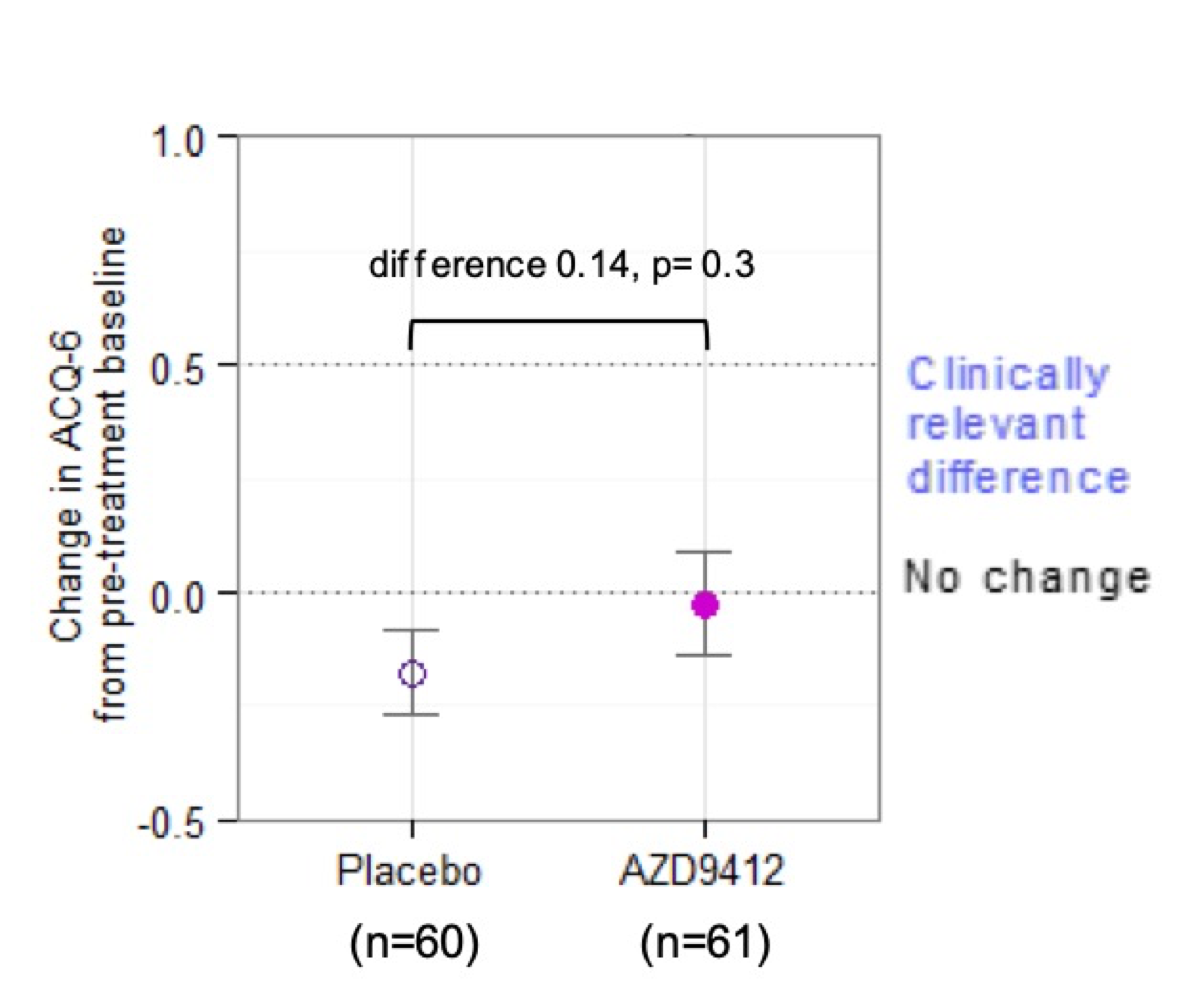

Supplement: Supplementary file 1 — Supplementary Material [file CEA-51-273-s001.docx]
